# Predictive Modeling of Vaccination Uptake in US Counties: A Machine Learning–Based Approach

**DOI:** 10.2196/33231

**Published:** 2021-11-25

**Authors:** Queena Cheong, Martin Au-yeung, Stephanie Quon, Katsy Concepcion, Jude Dzevela Kong

**Affiliations:** 1 School of Kinesiology University of British Columbia Vancouver, BC Canada; 2 Faculty of Science University of British Columbia Vancouver, BC Canada; 3 Faculty of Applied Science University of British Columbia Vancouver, BC Canada; 4 Africa-Canada Artificial Intelligence and Data Innovation Consortium Department of Mathematics and Statistics York University Toronto, ON Canada

**Keywords:** COVID-19, vaccine, public health, machine learning, XGBoost, SARS-CoV-2, sociodemographic factors, United States, sociodemographic, prediction, model, uptake

## Abstract

**Background:**

Although the COVID-19 pandemic has left an unprecedented impact worldwide, countries such as the United States have reported the most substantial incidence of COVID-19 cases worldwide. Within the United States, various sociodemographic factors have played a role in the creation of regional disparities. Regional disparities have resulted in the unequal spread of disease between US counties, underscoring the need for efficient and accurate predictive modeling strategies to inform public health officials and reduce the burden on health care systems. Furthermore, despite the widespread accessibility of COVID-19 vaccines across the United States, vaccination rates have become stagnant, necessitating predictive modeling to identify important factors impacting vaccination uptake.

**Objective:**

This study aims to determine the association between sociodemographic factors and vaccine uptake across counties in the United States.

**Methods:**

Sociodemographic data on fully vaccinated and unvaccinated individuals were sourced from several online databases such as the US Centers for Disease Control and Prevention and the US Census Bureau COVID-19 Site. Machine learning analysis was performed using XGBoost and sociodemographic data.

**Results:**

Our model predicted COVID-19 vaccination uptake across US counties with 62% accuracy. In addition, it identified location, education, ethnicity, income, and household access to the internet as the most critical sociodemographic features in predicting vaccination uptake in US counties. Lastly, the model produced a choropleth demonstrating areas of low and high vaccination rates, which can be used by health care authorities in future pandemics to visualize and prioritize areas of low vaccination and design targeted vaccination campaigns.

**Conclusions:**

Our study reveals that sociodemographic characteristics are predictors of vaccine uptake rates across counties in the United States and, if leveraged appropriately, can assist policy makers and public health officials to understand vaccine uptake rates and craft policies to improve them.

## Introduction

The COVID-19 pandemic has affected millions worldwide. The widespread impact of the disease has forced populations into lockdown and self-isolation, and to social distance from each other to mitigate the disease spread. As a result, many await the successful development of a COVID-19 vaccine to return to normality. However, even if one becomes readily available, enough people need to have access or be willing to receive the vaccine to achieve herd immunity [1]. Previous literature has indicated disparities in vaccination rates between sociodemographic groups, and such factors play a substantial role in the likelihood of seeking vaccination. For example, those with lower education and income level [2,3], and Black individuals [4] are less likely to get vaccinated. Thus, the purpose of the study aims to use machine learning classification algorithms to construct a model that can predict vaccine uptake for US counties using publicly available sociodemographic data. Using this, public health officials can develop targeted interventions for specific populations to promote vaccine uptake by forecasting future vaccine behaviors. With the recent development in technological methods, researchers’ use of machine learning methods to predict the likelihood of health behaviors has been on the rise. Previous studies have used XGBoost (extreme gradient boosting), a decision tree–based machine learning algorithm that uses a gradient-boosting framework, to build predictive models for vaccination uptake levels for influenza [5] and childhood immunizations [6]. Given the urgency of public health officials to encourage COVID-19 vaccination worldwide, such methods have substantial applicability in the current epidemiological context. Although many studies have investigated the impact of sociodemographic factors on vaccine uptake on a national level, research on a county level is scarce. Additionally, the use of data on smaller regions allows for a better understanding of local vaccine behaviors. This study seeks to fill this knowledge gap by incorporating a broad range of sociodemographic characteristics between US counties to predict COVID-19 vaccination uptake.

## Methods

### Sourcing the Data

Sociodemographic and socioeconomic data was collected from the US Department of Agriculture [7-9], the US Centers for Disease Control and Prevention [10], US Bureau for Labor Statistics [11], US Census Bureau COVID-19 Site [12-24], and Simple Maps US Counties Database [25]. In each database, data was collected for each US county and identified by its Federal Information Processing System (FIPS) code. This study included 2862 US counties out of 3007, and the counties were used based on the overlapping FIPS codes between data sets. The state of Texas was excluded as they did not release their COVID-19 vaccination data. The data set includes data collected between 2015 and 2021.

From these databases, 83 sociodemographic factors were collected and organized into 20 categories: education, ethnicity, income, employment, poverty, household size, population density, age, sex, disability status, access to technology, language spoken, health insurance, occupation, location, housing tenure, educational enrollment, grandparents taking care of grandchildren, access to income benefits, and working at home. Each category has between 1 and 14 subfactors. For example, the education category’s factors are the percentage of adults with less than a high school diploma, percent of adults with a high school diploma only, percent of adults completing some college or associate degree, and percent of adults with a bachelor’s degree or higher. A complete list of factors and their associated categories can be found in Table S1 in Multimedia Appendix 1. In addition, the percent of adults fully vaccinated against COVID-19 was found from the US Centers for Disease Control and Prevention [10]. The percentages are representative of vaccination data from May 21, 2021.

### Creating a Universal Model

XGBoost Regressor was used as the predictive modeling algorithm to create a supervised regression model. XGBoost was chosen over other traditional machine learning methods because it is a decision tree–based model. This particular method can closely mimic the adaptive and consequential nature of the human decision-making process. In other words, a decision tree–based model can mimic how humans consider the potential outcomes of their actions before making a decision. Thus, our model provides a more accurate real-life depiction of how certain sociodemographic factors lead to decisions to take the vaccine. Furthermore, from an analytical standpoint, XGboost prevents overfitting and brings performance improvements compared to other traditional machine learning methods (eg, linear regression, elastic net, and random forest) since it uses a more regularized model formalization. In short, regularization is the process of adding information to solve a problem without overfitting, a process where a model fits too closely to its training data [26].

Other than performance alone, XGBoost has demonstrated great accuracy over other methods. For example, a previous study comparing the accuracy of different predictive modeling algorithms shows that XGBoost shows the highest accuracy score compared to other methods such as logistic regression, naive Bayes classifier, decision trees, and random forest [27]. Furthermore, XGBoost has demonstrated to learn better tree structures over decision tree models that use gradient boosting since XGBoost uses Newton boosting instead [28].

Lastly, we chose XGBoost because of its previous track record in the competitive machine learning scene. For example, in 2015, when Kaggle published the 29 winning solutions on their blog, it was found that 17 solutions used XGBoost [26]. The data science platform has also interviewed many of their top-ranking competitors on several occasions, and when asked what their favorite machine learning algorithms were, four members who have ranked as number one responded with XGBoost [28]. The annual data mining and knowledge discovery competition KDDCup 2015 further elucidates the system’s prevalence, where the top 10 winning teams used XGBoost [26]. Examples of problems in these winning solutions include store sales prediction, ad click-through rate prediction, and hazard risk prediction [26]. The evident success of this method in solving myriads of real-life scenarios and problems demonstrates its effectiveness and versatility in predictive health modeling.

In conclusion, with the aforementioned factors, XGBoost is a highly effective, efficient, and robust machine learning method with many benefits toward the needs of our paper.

### Hypertuning Parameters for XGBoost

We used ExhaustiveGrid Search Cross-Validation (GridSearchCV) to perform five folds for each of the 384 permutations (totaling 1920 fits) to search for the optimal parameters to use in our XGBoost model to provide the highest accuracy in predicting vaccination uptake, specifically for our particular data sets. The learning rate represents how quickly an error is corrected from each tree. The max_depth determines the maximum depth a tree is allowed to grow during each boosting round. The min_child_weight is the minimum sum of instance weight needed in a child.

The subsample parameter randomly sets how much some of the training data is sampled prior to growing trees to prevent overfitting. The colsample_bytree parameter is the subsample ratio of columns when constructing each tree, again to prevent overfitting. N_estimators represent the number of trees to grow for the model. The range of parameters that we searched for the best fit is in Table S2 in Multimedia Appendix 1. The parameters we used in our model after computational fit (grid search best score of 0.5523) are illustrated in Table 1.

**Table 1:** Selected tuning parameters that were chosen after computational fit (grid search best score of 0.5523).

Parameters	Ranges
Learning rate	0.01
max_depth	9
min_child_weight	3
Subsample	0.7
colsample_bytree	0.7
n_estimators	1000

### Evaluating the Model’s Accuracy and Error

We adopted the use of k-fold cross-validation using the Scikit-learn package in Python (Python Software Foundation) to determine our accuracy score. A cross-validation method was chosen due to its ability to estimate the skill of a machine learning model based on unseen data. This can provide an estimate on how the model performs when used to make predictions on data not used during the training of our model—our accuracy percentage. The k-fold cross-validation uses a method where the cross-validation method is split into several groups that a given data sample is to be split into, defined by k. We chose a k value of 10, as this value is shown to have test error rate estimates that do not have high bias or high variance [29]. The final percentage accuracy representation produced by our k-fold cross-validation analysis is the percentage alignment with vaccination rates (percentage of population) in the test set with the predicted values.

We leveraged the root mean squared error (RMSE) to calculate our model’s error, as RMSE is measured in the same units as the target variable (vaccination uptake percentage), providing us with a better interpretation and understanding of the errors of our models. RMSE measures the square root of the sum, for the vaccination uptake, of the square of the difference between the predicted (*y_j_*) and actual vaccination uptake (*ŷ_j_*), divided by the number of counties.



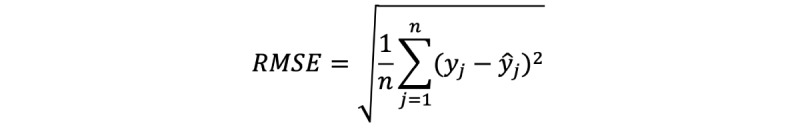



### Evaluating a Feature’s Importance

We used XGBoost’s built-in feature importance, permutation analysis, and SHAP to understand how each feature drives our model’s prediction score. Comparing these three aggregated methods allows us to understand how covariates contribute to the model fitting and the importance of the features we used for our vaccination model, rather than simply using one method. To avoid bias due to specific feature categories containing more factors, we used Breiman’s permutation-based measures to assess the importance of a feature by calculating the degree of increase in the model’s prediction error after their values are permuted, otherwise known as randomized ablation [30]. This approach provided us with a way to investigate the independent predictive power of each category without building separate machine learning models for each feature category to do so. We performed permutation analysis using the Python Scikit-learn library. The SHAP library provided a game-theoretic approach to determine an overview of which features are most important for a model by plotting the absolute mean SHAP value in a bar graph [31]. Lastly, XGBoost’s built-in feature importance calculated how important a feature is from its corresponding score. We then aggregated the top five features from these three methods; then, we selected all features demonstrated in the three feature importance analysis methods as our final list of selected important features driving vaccination uptake.

## Results

Using XGBoost’s regressor function hypertuned with custom parameters, our model predicted the percentage of COVID-19 vaccination uptake per county with an accuracy of 62%. This accuracy score was calculated based on the k-fold cross-validation average score.

Our model demonstrated relatively low errors using RMSE analysis, where it demonstrated a 0.08% vaccination uptake percentage error compared to the test model and 0.05% vaccination uptake percentage error compared to the actual model.

**Figure 1:**
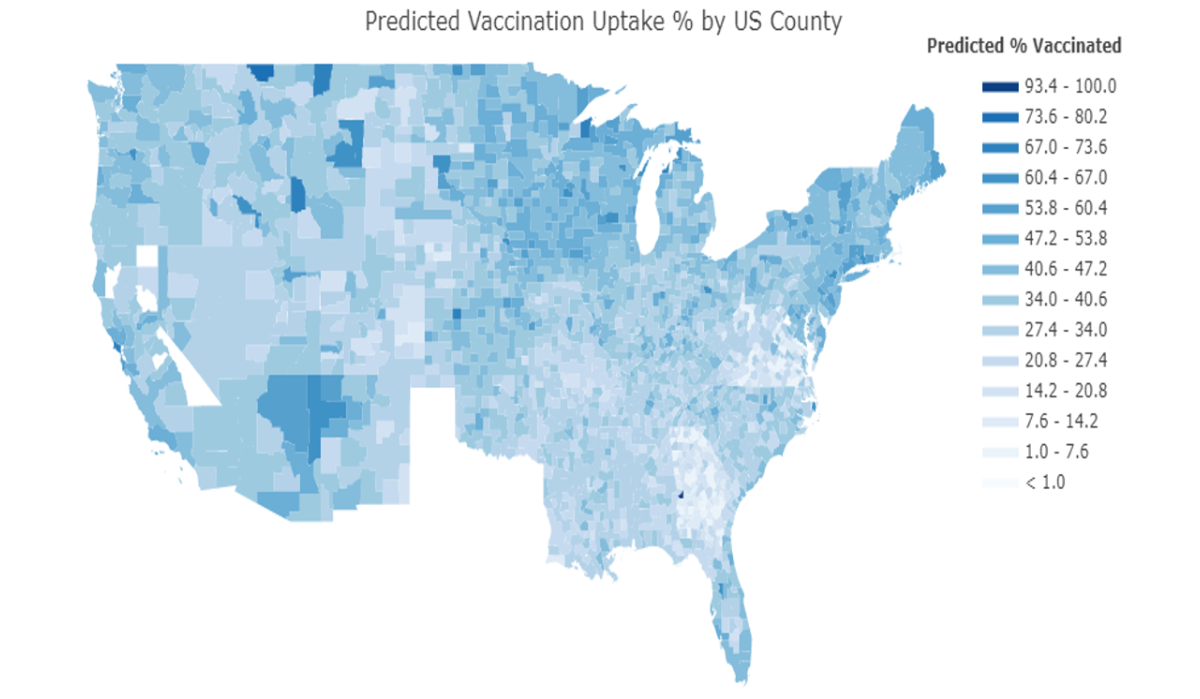
Predicted vaccination uptake percentage by US counties. White areas represent areas with no vaccination uptake data.

The choropleth map of US counties shown in Figure 1 demonstrates our machine learning model’s predicted percentage of vaccine uptake. White areas on the choropleth maps represent areas with no vaccination uptake data or other missing sociodemographic data.

**Figure 2:**
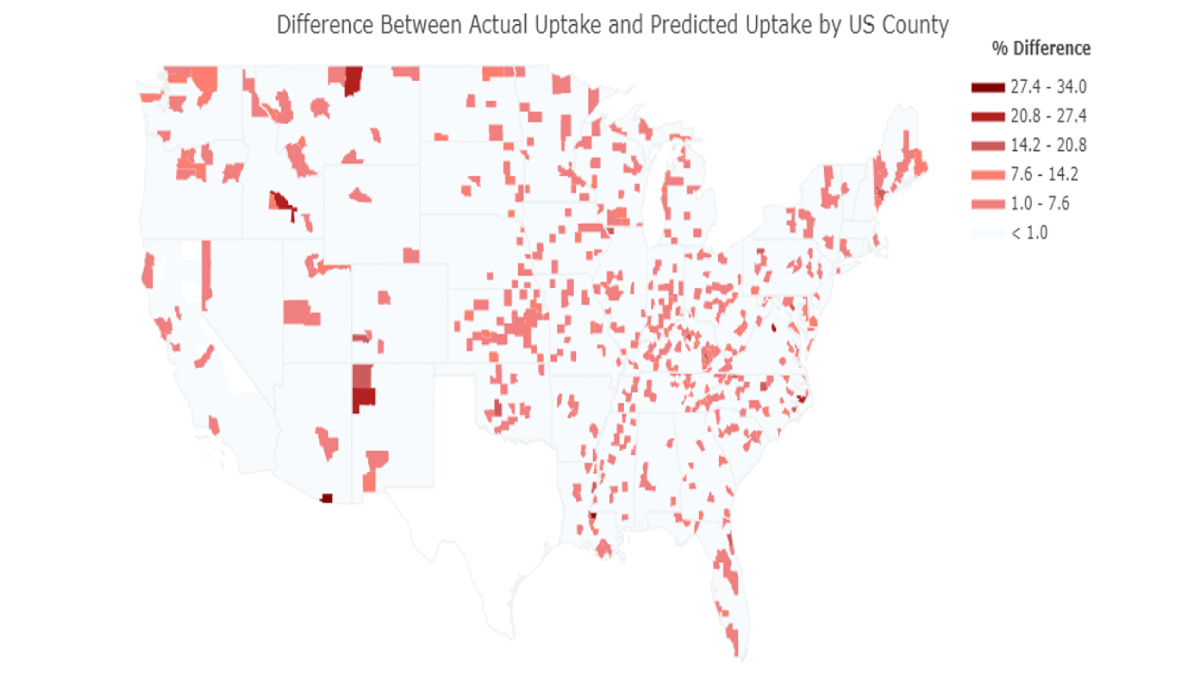
Accuracy of the model in predicting actual vaccination uptake by US county. The darkest shades of red represent lower prediction accuracy, with white representing the highest prediction accuracy.

In Figure 2, the choropleth map of US counties shows the difference between actual uptake and predicted uptake, and highlights counties where our model was less accurate in predicting vaccination uptake.

**Figure 3:**
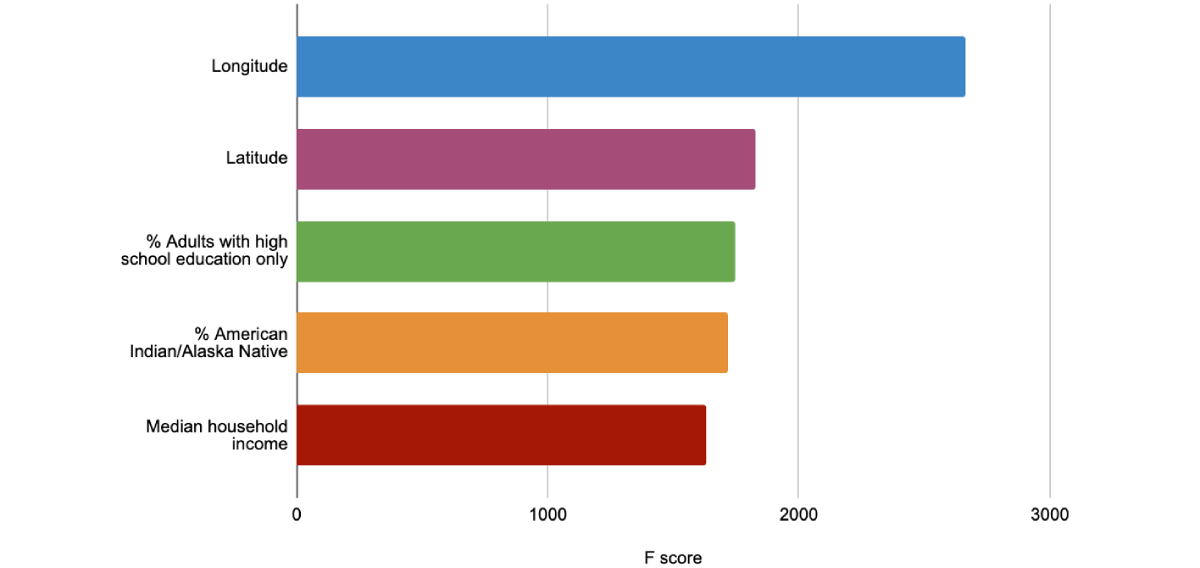
The top five identified sociodemographic factors to predict vaccination uptake by XGBoost's built-in feature importance analysis function.

Figure 3 highlights the top five features generated from XGBoost’s built-in plot importance function in ranking order of importance. The top five features that were identified to drive XGboost’s predictive model were geographic location (longitude, latitude), adults with less education (percent of adults with a high school diploma only), indigenous population (percent non-Hispanic American Indian/Alaska Native), and median household income (median household income percent of the state). The top 25 features generated from XGBoost’s built-in plot importance can be found in Table S3 in Multimedia Appendix 1.

**Figure 4:**
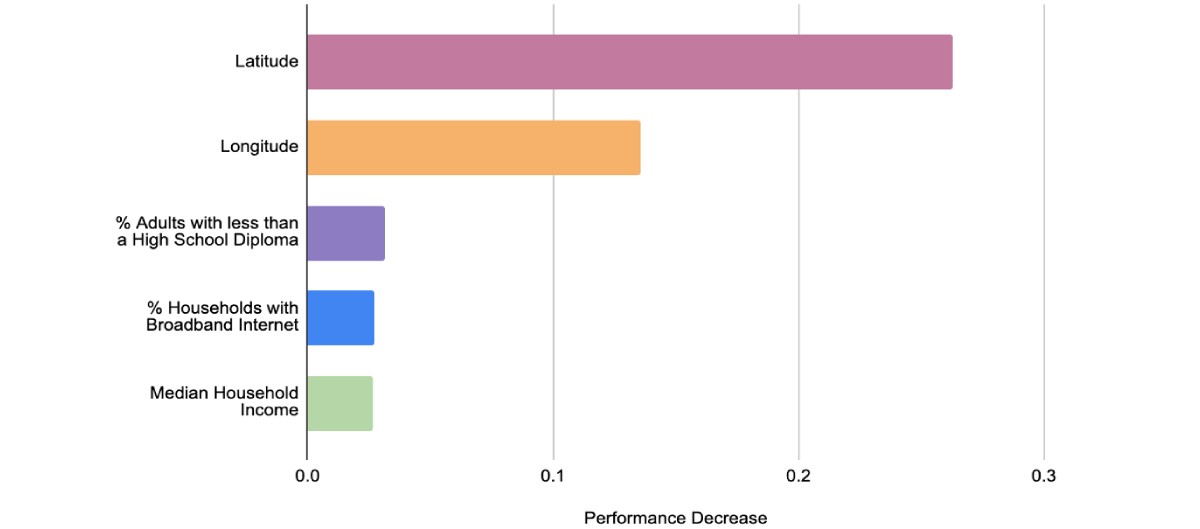
The top five identified sociodemographic factors to predict vaccination uptake found by permutation analysis.

Figure 4 illustrates the top five features generated by the Python Scikit-learn package permutation importance function in ranking order of importance. The most notable features found are geographic location (longitude, latitude), education (percent of adults with less than a high school diploma), online access (households with broadband internet), and income (median household income percent of the state). The top 25 features generated by this approach can be found in Table S4 in Multimedia Appendix 1.

**Figure 5:**
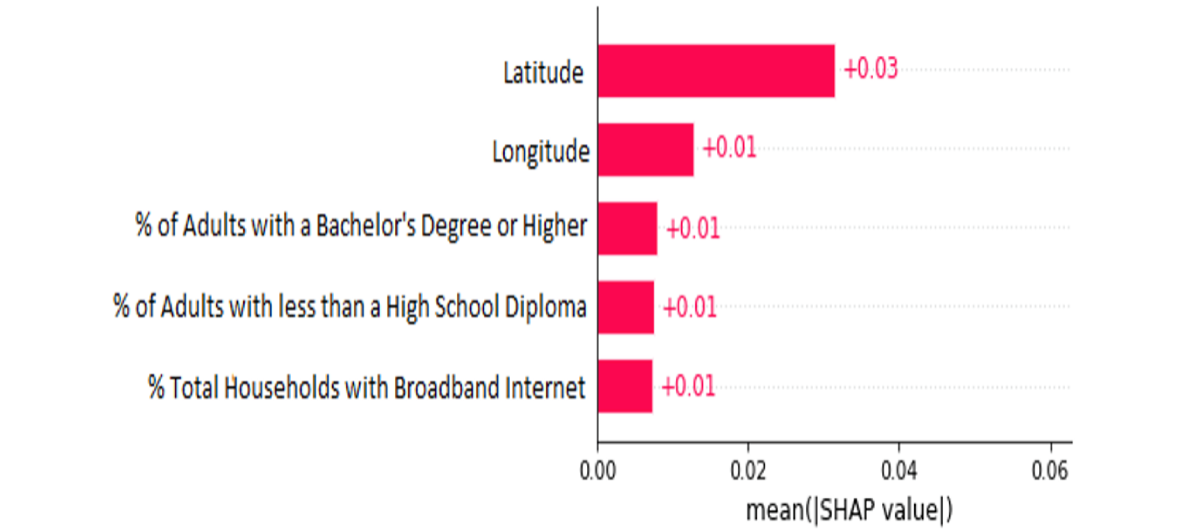
The top five identified sociodemographic factors that drive the model's prediction for vaccination uptake found by SHAP.

Figure 5 demonstrates the top five features generated by SHAP in ranking order of importance. The top significant features that SHAP found to drive our predictive model based on SHAP were geographic location (longitude, latitude), education level (percent of adults with less than a high school diploma, percent of adults with a bachelor’s or higher), and online access (households with broadband internet). The ranking influence of the remaining features generated by this approach can be found in Figure S2 in Multimedia Appendix 1.

## Discussion

Our XGBoost model scored a 62% accuracy score in predicting vaccination uptake based on 83 sociodemographic factors in this study. We also determined that geographic location, education, household accessibility to broadband internet, median household income, and ethnicity were the six main factors driving our model’s prediction across analyses done by XGBoost, permutation analysis, and SHAP.

### Accuracy of Predictive Modeling

Our machine learning model scored a k-fold cross-validation accuracy score of 62%, representing the model’s ability to accurately predict vaccination uptake based on sociodemographic factors. The accuracy score alone does not provide sufficient information regarding the mechanism by which a sociodemographic factor impacts a population. However, the machine learning model can produce a choropleth with individualized percentage accuracy scores per county, where health authorities and governing bodies can use these percentage scores to visualize areas with lower than average vaccination uptake. Therefore, the results of this machine learning analysis can advise those in charge of mitigating the pandemic in identifying at-risk areas in need of targeted vaccination campaigns.

In Figure S1 in Multimedia Appendix 1, we plotted the actual vaccine uptake per county on a choropleth of the United States. Darker regions represent areas with higher vaccination rates, while lighter regions represent areas with lower vaccination rates. Figure 1 shows our machine learning model’s predicted vaccination rate per county. When comparing the two plotted maps, the observed differences between the actual and predicted models were less distinct, denoting evidence of our model’s ability to visualize vaccination uptake accurately. The state of Texas was excluded from our map, as they did not release their COVID-19 vaccination uptake data.

However, our current model does not consider additional nonsociodemographic factors such as policies enacted by local governments, political views in local areas, and citizens’ general behavior, limiting our model’s accuracy score. Therefore, to encompass vaccination rate and increase the accuracy score of our model, we must also view nonsociodemographic factors, as they can also substantially drive an individual’s decision to take the vaccine.

To further evaluate the accuracy of our regression model, we generated a choropleth identifying the counties where our model had difficulties or ease in predicting vaccination (Figure 2). As evident, there are few counties where the model had low prediction accuracy—the worst being a 27.4% to 34.0% difference between the predicted and actual vaccination uptake. The two counties where the regression model struggled with predicting the most are Santa Cruz, Arizona (AZ) and West Feliciana, Los Angeles (LA). As well, other counties that the model yielded a 20.8% to 27.4% difference between predicted and actual vaccination uptake are McKinley, New Mexico (NM); Blaine County, Montana (MT); and Blaine County, Idaho (ID).

The generated prediction accuracy choropleth in Figure 2 can be helpful for public health authorities since it identifies which counties the model was accurate or inaccurate in predicting actual vaccination uptake. With this knowledge, officials and health governing bodies can better understand and decide which areas they need to target their efforts toward. In addition, the counties that our analysis has identified to have low prediction accuracies, such as Santa Cruz, AZ, can be an avenue for future research to investigate why there was a high prediction error in those particular counties.

As well, the predictability accuracy can allow public health authorities in future pandemics to predict more precisely an estimated vaccination uptake based solely on sociodemographic factors. Thus, targeting ahead of time areas that should prioritize education on vaccination safety and why it is essential to receive the vaccine.

### Feature Importance

The significance of the top identified sociodemographic features by our model—location, education, ethnicity, income, and access to the internet—provide a vivid portrayal of the current social climate in the United States and is calculated by using XGBoost’s built-in feature importance (Figure 3), permutation analysis (Figure 4), and SHAP (Figure 5).

Based on the following three methods in determining feature importance, we determined that geographic location has the most influence on our prediction model, with both latitude and longitude ranking first or second, respectively. As well, the third feature is primarily dominated by an educational-based factor (school level).

Our feature importance analyses ranking location and education so highly also provides a more comprehensive look at other aspects of American features that are prominent presently, such as lack of education and political divide in rural areas compared to more populated areas. Our evidence supports that these sociodemographic factors significantly influence disparities in access to health resources and must continue to be the focus of public and government efforts to decrease the gap.

#### Longitude and Latitude

Based on our feature importance analysis methods, our machine learning model determined that longitude and latitude were the top two most crucial factors across all three methods, suggesting that geographic location plays the most prominent role in vaccine uptake. There may be an interaction term between longitude and latitude, given there may be a three-way relationship between longitude, latitude, and vaccine uptake. In past research studies, individuals residing in the western United States were more likely to refuse their children’s vaccinations [32], and a recent study found that COVID-19 vaccination coverage is lower in rural counties [33]. In addition, population density, which ties in closely with the significance of a geographic region, ranked ninth in importance on our factors list. With this in mind, our results demonstrate that geographic location has a clear role in driving vaccination uptake. However, our results do not precisely determine the direct relationship between how geographic location influences vaccine uptake. Therefore, future research could explore the specific implications of location and living in rural areas to determine where additional COVID-19 vaccination centers can be opened.

#### Education

Although this study explored several educational factors related to level of education and educational enrollment, the factor with the highest rank of importance was the percent of adults with a high school diploma only. This factor ranked third in importance, and two other educational factors ranked in the top 8 factors.

Previous research has indicated a relationship between vaccine uptake and education. In a study, individuals who had attained higher levels of education were more likely to accept vaccines as safe [34] and vaccinate individuals in their care, such as their children [35]. As our results do not determine the direct relationship between education and vaccine uptake, it cannot be concluded that level of education increases or decreases vaccine uptake. However, our results demonstrate that educational groups separated by secondary school attainment could be an important factor in determining whether an individual will receive the COVID-19 vaccine. Therefore, future research could explore this specific factor and how it impacts vaccine uptake.

#### Ethnicity

Despite being twice as likely to die from COVID-19 [36], there is consistent evidence that ethnic minority groups are less likely to be vaccinated for the virus (eg, [37]) compared to their White counterparts. Factors that have been explained to drive this inequality include differences in vaccine hesitancy [38], attitudes toward vaccines [39], and trust in distributing pharmaceutical companies between ethnic groups [39]. In our findings, ethnicity is the fourth most important factor associated with vaccination uptake, providing evidence that an individual’s cultural background is substantially associated with whether or not they receive vaccination. Thus, these findings provide support that there is a need for the development of special efforts to target historically marginalized populations in vaccination campaigns and increase vaccination rates among those groups with low uptake.

#### Median Household Income

In previous studies, individuals from lower-income households were less confident in the importance of vaccines [40] while also being more vulnerable to the impacts of COVID-19 [35]. Household income also relates closely to socioeconomic status, and individuals belonging to higher socioeconomic status groups are more likely to receive vaccines [41]. This combination of factors stemming from income and financial status may impact an individual’s ability and willingness to receive the COVID-19 vaccine. Our finding that household income is an important factor in vaccine uptake is consistent with previous literature. However, our results do not determine if lower or higher income households are more likely to receive the COVID-19 vaccine. Therefore, more research is needed to explore the relationship between these two factors. However, given the previous evidence that individuals from lower-income groups are less likely to receive vaccinations, vaccine campaigns could further target their efforts toward lower-income groups to ensure that more individuals have access to vaccines and have confidence in them.

#### Internet Accessibility

Our feature importance analyses also revealed that household access to broadband internet was significant in predicting vaccination uptake. This is an important find, as digital technology played a substantial role during the COVID-19 pandemic in communicating health information from administrations to the public, aiding disease surveillance, and developing mobile health apps [42]. In addition, the convenience of social media enables many communities, particularly historically marginalized groups, to access critical COVID-19 data and information more readily and easily. Thus, many public health agencies sought online appointment platforms to assist with their vaccination booking processes during the pandemic. This means that having direct access to the internet can play a role in determining whether an individual can receive a vaccine. However, being knowledgeable and literate on how to use it may possibly be even more pivotal.

Published studies have reported racial and educational differences in digital literacy. For example, a US Department of Education report posits that Black people were twice as likely to be digitally illiterate than their White counterparts [43]. An individual’s level of formal education also affects their knowledge of computer literacy [44]. Such factors may contribute heavily to the ability of individuals to identify misinformation on the internet and the desire to get a COVID-19 vaccination—otherwise known as the degree that they are vaccine hesitant. The World Health Organization has cited vaccine hesitancy as one of the top 10 threats to global health, as this delay in acceptance threatens the process of tackling widespread viruses and diseases [45].

With the study’s finding that asserts that household access to the internet is primary in predicting one’s vaccination uptake, we can bring forth awareness to public health officials about the importance of centering their efforts in providing greater accessibility to broadband internet in communities that may not have widespread internet use and teach them about digital literacy. This will enable those communities with the skills to critically interpret the vast proliferation of health information during this pandemic. Doing so could potentially help certain groups alleviate vaccine concerns, better understand the scientific rationale behind vaccines, and recognize misinformation when they encounter it.

### Conclusions

Although in the United States, COVID-19 cases are moving toward a downward trajectory and counties are beginning to fully reopen, the study is important for future pandemics or even if new variants may require new vaccination development. Furthermore, by constructing a model that can predict future vaccine behaviors in US counties, we can better advise public health authorities in advance, allowing them to prepare areas of vaccination campaign focus more efficiently and effectively.

## Appendix

Multimedia Appendix 1 Supplementary information.

## Abbreviations

FIPS: Federal Information Processing System
RMSE: root mean squared error
