# Supplementary material for: Predictive Modeling of Vaccination Uptake in US Counties: A Machine Learning–Based Approach
Source: J Med Internet Res. 2021 Nov 25;23(11):e33231. doi: 10.2196/33231 (PMC8623305; doi:10.2196/33231)
Supplement: Multimedia Appendix 1 [file jmir_v23i11e33231_app1.docx]

*Supplementary Information for*

*Predictive Modeling of Vaccination Uptake in U.S. Counties: A Machine Learning-based Approach*

Queena Cheong^1†^, Martin Au-yeung^2†^, Stephanie Quon^3^, Katsy Concepcion^2^, Jude Dzevela Kong^4*.^

1 School of Kinesiology University of British Columbia Vancouver CA

2 Faculty of Science University of British Columbia Vancouver CA

3 Faculty of Applied Science University of British Columbia Vancouver CA

4 Africa-Canada Artificial Intelligence and Data Innovation Consortium (ACADIC), Department of Mathematics and Statistics York University Toronto, ON

^†^ Co-first authors

*Corresponding author: Jude Dzevela Kong
*Email*: [jdkong@yorku.ca](mailto:jdkong@yorku.ca)

| *Category* | *Factors* |
| --- | --- |
| Education | Percent of adults with less than a high school diploma, 2015-19 |
|  | Percent of adults with a high school diploma only, 2015-19 |
|  | Percent of adults completing some college or associate's degree, 2015-19 |
|  | Percent of adults with a bachelor's degree or higher, 2015-19 |
| Ethnicity | Percent Hispanic |
|  | Percent non-Hispanic American Indian/Alaska Native |
|  | Percent non-Hispanic Asian |
|  | Percent non-Hispanic Black |
|  | Percent non-Hispanic Native Hawaiian/Pacific Islander |
|  | Percent non-Hispanic White |
| Income | Median household income as a percent of state total, 2019 |
|  | Median household income, 2019 |
| Employment | Unemployment rate, 2019 |
| Poverty | Estimated percent of people ages 0-17 in poverty 2019 |
|  | Estimated percent of people of all ages in poverty 2019 |
|  | Estimated percent of related children ages 5-17 in families in poverty 2019 |
|  | Percent households: income below poverty level |
|  | Percentage of people whose income in the past 12 months is below the poverty level |
|  | Percent population with income to poverty ratio < 0.50 |
|  | Percent population with income to poverty ratio 0.50-0.99 |
|  | Percent population with income to poverty ratio 1.00-1.24 |
|  | Percent population with income to poverty ratio 1.50-1.84 |
|  | Percent population with income to poverty ratio 1.85-1.99 |
|  | Percent population with income to poverty ratio 2.00+ |
|  | Percentage of families whose income in the past 12 months is below the poverty level with female householder, no husband present) - With related children of the householder under 18 years |
|  | Percentage of families whose income in the past 12 months is below the poverty level with female householder, no husband present) - With related children of the householder under 5 years only |
|  | Percentage of people whose income in the past 12 months is below the poverty level - 65 years and over |
|  | Percentage of people whose income in the past 12 months is below the poverty level - Related children of the householder under 5 years |
| Household Size | Average household size of owner-occupied unit, 2020 |
|  | Average household size of rented-occupied unit, 2020 |
|  | Average household size, 2020 |
| Population Density | Population density, 2020 |
|  | Population |
| Age | Median age |
|  | Percent 18 years and over |
|  | Percent 5 to 9 years |
|  | Percent 65 to 74 years |
|  | Percent 65 years and over |
|  | Percent 75 to 84 years |
|  | Percent 85 years and over |
|  | Percent under 5 years |
| Assigned Sex | Percent male |
|  | Percent female |
| Disability Status | Percent households with 1+ persons with disability |
|  | Percent total civilian noninstitutionalized population - with a disability 65 and over |
|  | Percent total civilian noninstitutionalized - with a disability |
| Access to Technology | Percent of households with Internet at home |
|  | Percent Total households with a computer |
|  | Percent Total households with a broadband Internet subscription |
| Language Spoken | Percent of households with Limited English speaking status |
| Health Insurance | Percent of civilian noninstitutionalized population with health coverage |
|  | Percent of population with no health coverage |
|  | Percent of total civilian noninstitutionalized population - No health insurance coverage |
|  | Percent of total civilian noninstitutionalized population - With health insurance coverage |
|  | Percent of total civilian noninstitutionalized population - With private health insurance |
|  | Percent of total civilian noninstitutionalized population - With public coverage |
| Occupation | Percent of civilian employed population 16 years and over in educational instruction, and library occupations |
|  | Percent of civilian employed population 16 years and over in food preparation and serving-related occupations |
|  | Percent of civilian employed population 16 years and over in healthcare practitioners and technical occupations |
|  | Percent of civilian employed population 16 years and over in healthcare support occupations |
|  | Percent of civilian employed population 16 years and over in natural resources, construction, and maintenance occupations |
|  | Percent of civilian employed population 16 years and over in production, transportation and material moving occupations |
|  | Percent of civilian employed population 16 years and over in protective service occupations |
| Location | Latitude |
|  | Longitude |
| Housing Tenure | Average household size of owner-occupied unit |
|  | Average household size of renter-occupied unit |
|  | Percent renter-occupied units |
| Educational Enrollment | Percent population enrollment in - College |
|  | Percent population enrollment in - College or graduate school |
|  | Percent population enrollment in - Elementary school (grades 1-8) |
|  | Percent population enrollment in - Grad/Prof |
|  | Percent population enrollment in - Grade 1-4 |
|  | Percent population enrollment in - Grade 5-8 |
|  | Percent population enrollment in - Grade 9-12 |
|  | Percent population enrollment in - High school (grades 9-12) |
|  | Percent population enrollment in - Kindergarten |
|  | Percent population enrollment in - Nursery, Preschool |
| Grandparents | Percent grandparents responsible for grandchildren |
| Access to Income Benefits | Percent total households - With Supplemental Security Income |
|  | Percent total households - With cash public assistance income |
|  | Percent total households - With Food Stamp/SNAP benefits in the past 12 months |
| Worked at Home | Percent total population - Worked at home |

*Table S1*: Full list of categories and features

| *Parameters* | *Ranges* |
| --- | --- |
| learning rate  min_child_weight  max_depth  subsample  colsample_bytree  n_estimators | [0.01, 0.1]  (3,11,2)  [1,3,5]  [0.5,0.7]  [0.5,0.7]  [100,200,500,1000] |

*Table S2.* Tuning parameter ranges tested for best computational fit.

| Rank | F score | Feature |
| --- | --- | --- |
| 1 | 2664 | Longitude |
| 2 | 1827 | Latitude |
| 3 | 1747 | Percent of adults with a high school diploma only, 2015-19 |
| 4 | 1720 | Percent non-Hispanic American Indian/Alaska Native |
| 5 | 1633 | Median household income percent of state (2019) |
| 6 | 1530 | Percent of adults with less than a high school diploma, 2015-19 |
| 7 | 1518 | Percent of adults completing some college or associate's degree, 2015-19 |
| 8 | 1505 | Percent non-Hispanic Native Hawaiian/Pacific Islander |
| 9 | 1453 | Population density, 2020 |
| 10 | 1442 | Percent of adults with a bachelor's degree or higher, 2015-19 |
| 11 | 1410 | Percent male |
| 12 | 1410 | Unemployment rate, 2019 |
| 13 | 1379 | Percent Hispanic |
| 14 | 1360 | Percent of civilian employed population 16+ in protective service occupations |
| 15 | 1353 | Percent non-Hispanic White |
| 16 | 1339 | Percentage of people whose income in the past 12 months is below the poverty level - Related children of the householder under 5 years |
| 17 | 1296 | Percent non-Hispanic Black |
| 18 | 1262 | Average household size of renter-occupied unit |
| 19 | 1221 | Percent of civilian employed population 16 years and over in production, transportation and material moving occupations |
| 20 | 1220 | Percent non-Hispanic Asian |
| 21 | 1189 | Percent under 5 years |
| 22 | 1165 | Percent grandparents responsible for grandchildren |
| 23 | 1160 | Percent total civilian noninstitutionalized population - with a disability 65 and over |
| 24 | 1147 | Percent population enrollment in - Nursery, preschool |
| 25 | 1143 | Percent total population - Worked at home |

*Table S3.* Ranked feature importance using XGBoost feature importance.

| Rank | Performance Impact | Feature |
| --- | --- | --- |
| 1 | 0.2630466861138317 | Latitude |
| 2 | 0.13544059670007533 | Longitude |
| 3 | 0.03134350940047288 | Percent adults fully vaccinated against COVID-19 |
| 4 | 0.027241859589174043 | Percent of population with no health insurance coverage |
| 5 | 0.026860268206766326 | Median household income as a percentage of the state average |
| 6 | 0.023367673388392407 | Percent total households with a broadband Internet subscription |
| 7 | 0.01489836280546446 | Percent of total civilian noninstitutionalized population |
| 8 | 0.014769607470627477 | Average household size of renter-occupied unit |
| 9 | 0.014366414704860442 | Percent of adults completing some college or associate's degree, 2015-19 |
| 10 | 0.012920434444535167 | Percent 85 years and over |
| 11 | 0.011189871624736702 | Percent total civilian noninstitutionalized population  - with a disability |
| 12 | 0.011173264025090047 | Percent total households with a computer |
| 13 | 0.01010649734595388 | Percent of civilian employed population 16 years and over in natural resources, construction, and maintenance occupations |
| 14 | 0.009166842560115374 | Percent of total civilian noninstitutionalized population - With public coverage |
| 15 | 0.008787855194352167 | Population |
| 16 | 0.006548700443273603 | Unemployment rate, 2019 |
| 17 | 0.004994518100647882 | Percent of civilian employed 16 years and over in healthcare practitioners and technical occupations |
| 18 | 0.004478135701747199 | Percent of total civilian noninstitutionalized population - With private health insurance |
| 19 | 0.004407073722088217 | Percent total civilian noninstitutionalized population - with a disability 65 and over |
| 20 | 0.0041645158871867595 | Percent of adults completing some college or associate's degree, 2015-19 |
| 21 | 0.0.004035050188122868 | Percent of total civilian noninstitutionalized population - With health insurance coverage |
| 22 | 0.0035768696780793087 | Percent Hispanic |
| 23 | 0.0035148919946434544 | Percent non-Hispanic Asian |
| 24 | 0.003454273625803883 | Percent non-Hispanic White |
| 25 | 0.003383120160398945 | Average household size of owner-occupied unit |

Table S4. Ranked feature importance using the Python scikit-learn’s permutation feature importance.


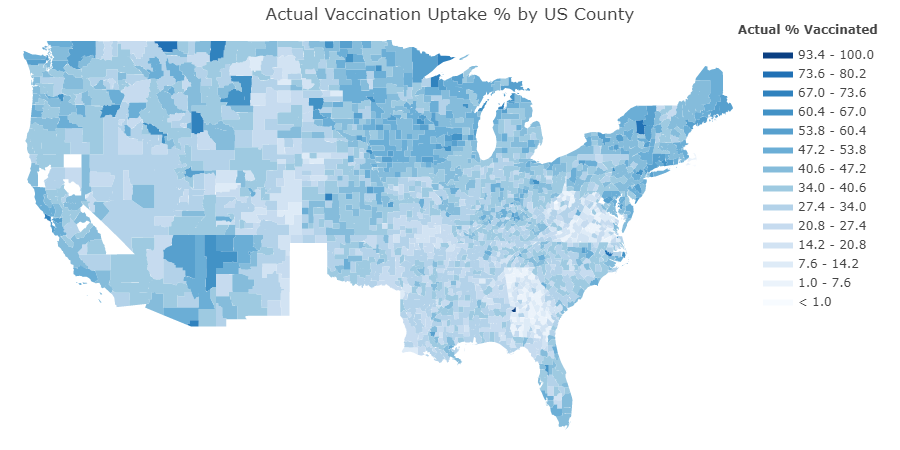


Figure S1. Actual vaccination uptake percentage by US counties.


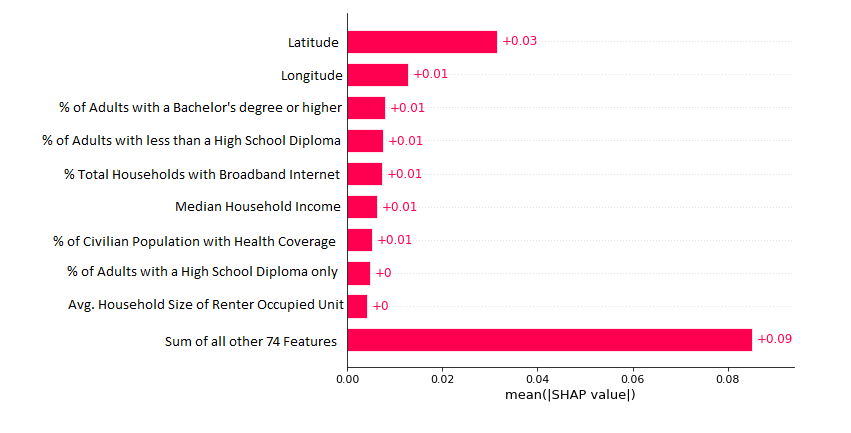


Figure S2. Full list of identified sociodemographic factors that predict vaccination uptake by SHAP.
